# Quantitative measurement and comparison of breakthroughs inside the gas diffusion layer using lattice Boltzmann method and computed tomography scan

**DOI:** 10.1038/s41598-024-60148-w

**Published:** 2024-04-26

**Authors:** Hossein Pourrahmani, Milad Hosseini, Hamza Moussaoui, Emad Oveisi, Majid Siavashi, Jan Van Herle

**Affiliations:** 1 Group of Energy Materials, École Polytechnique Fédérale de Lausanne, 1951 Sion, Switzerland; 2 School of Mechanical Engineering, Iran University of Science and Technology, Tehran, Iran; 3 Interdisciplinary Centre for Electron Microscopy (CIME), École Polytechnique Fédérale de Lausanne (EPFL), Lausanne, Switzerland

**Keywords:** Proton exchange membrane fuel cell (PEMFC), Gas diffusion layer (GDL), Computed tomography (CT) scan, Lattice Boltzmann method (LBM), Water columns (breakthrough), Mechanical engineering, Chemical engineering, Fuel cells

## Abstract

In Proton Exchange Membrane Fuel Cells (PEMFCs), the presence of residual water within the Gas Diffusion Layer (GDL) poses challenges during cold starts and accelerates degradation. A computational model based on the Lattice Boltzmann Method (LBM) was developed to consider the capillary pressure inside the PEMFC and to analyze the exact geometries of the GDLs, which were obtained using the Computed Tomography scan. The novelty of this study is to suggest a methodology to compare the quantitative water removal performance of the GDLs without long-term experimental testing. Two different samples of GDLs were considered, pristine and aged. The results of quantitative measurements revealed the amount of water columns (breakthroughs) inside each sample. Considering the volume of 12,250,000 µm^3^ for each sample, the pristine and the aged samples are prone to have 774,200 µm^3^ (6.32%) and 1,239,700 µm^3^ (10.12%) as water columns in their porous domain. Micro-structural properties such as connectivity, mean diameter, effective diffusivity, etc. were also compared to observe the impacts of aging on the properties of the GDL.

## Introduction

The harmful environmental impacts of fossil fuels and the low efficiency of combustion engines have resulted in finding alternative fuels^1^. In this regard, methanol, ethanol, bio-fuels, ammonia, hydrogen, etc. have been suggested as possible candidates^2^. Among the mentioned alternative fuels, hydrogen has been suggested as the most promising option for a wider spectrum of usages^3^. This means that hydrogen production methodologies should be developed to provide the required demand of fuel for different industries^4^. Hydrogen can be directly used in fuel cells to generate electricity, reach net zero carbon emissions, and act as a catalyst in ammonia/methanol production^5^.

As a promising technology to facilitate the transition from fossil fuels to hydrogen, fuel cells are proposed^6^. Among the introduced fuel cell variants, the Proton Exchange Membrane Fuel Cell (PEMFC) is considered the most efficient type in low-temperature operations^7^. To accelerate the commercialization of the PEMFCs, improving the water/thermal management, increasing the durability, and reducing the costs are essential^8^. In the long-term operation of the PEMFCs, flooding (the excessive accumulation of the water that enlarges the mass transport losses) or drying out of the membrane should be prevented, hence the operation of the cell in the temperature range of 65–85 ^∘^, and using a gas diffusion layer (GDL) is suggested^9^.

The GDL removes the excess liquid water from the catalyst layers (CL) and transfers the reactant gases from the flow fields^10^. In order to improve the water removal characteristic of the GDL, polytetrafluoroethylene (PTFE) is being used to improve the hydrophobicity of the utilized carbon fibers in the GDL and to provide a higher water removal^11^. Additionally, a microporous layer (MPL), which has similar characteristics as GDL except for having lower porosities, is being assembled between the GDL and the CL to prevent the remaining liquid water inside the cell, hence preventing the formation of ice in sub-zero temperatures^12^ (e.g. operation of the fuel cell electric vehicles in the winter).

Although the existence of the GDL and MPL in the PEMFC stacks improves water management, the long-term performance of the PEMFC degrades these two layers and results in flooding of the CL and membrane^13^. The degradation mechanisms for the PEMFC are either mechanical or electrochemical^14^. The compression, dissolution, erosion, and freeze/thaw cycling are the most renowned mechanical degradations for the PEMFCs while carbon corrosion and radical attack are the most common electrochemical types of degradation for the GDL and MPL^15^. Due to the high expenses and time-consuming procedures of the *In-Situ* degradation tests, *ex-Situ* Accelerated Stress Tests (ASTs) such as immersing in oxidizing baths, compression, and freeze-thaw cycling are being widely used. However, the existing assumptions and conditions of the *ex-Situ* experiments may not be correct in the real conditions and demand modifications^16^. In this regard, the usage of simulations and neural computing can be vaporized^17^.

Simulation methods have proved to have high precision and accuracy in different scales^18^. In macro- and meso- scales studies, commercial softwares use the energy, momentum, and mass principal equations to visualize the fluid flow in different phases^19^. Niu et al.^20^ developed a three-dimensional fluid dynamic study using the Volume of Fluid (VOF) model to analyze the impacts of PTFE loadings and the water saturation in GDL on the water removal behavior. It was figured out that the air-drying GDL leads to increased PTFE loadings near the cathode inlet and decreases the water absorption at the inlet. Although the VOF model is efficient to evaluate the performance of the system at the macro-scale, a validation of the VOF results should be developed with the LBM or experimental results.

It is believed that fluid simulations based on principal conservative equations, which are being used in commercial softwares like ANSYS and COMSOL, are not competitive enough to deal with complex boundaries, and to include microscopic interactions^21^. It is possible to improve the simulation results through User Defined Functions (UDFs) in the available commercial software^22^. However, the usage of the Lattice Boltzmann Method (LBM)^23^, which is based on kinetic energy between the particles rather than the principal conservative equations, will facilitate the simulations of multi-phase transport phenomena in the pore-scales due to the high accuracy/precision in all levels of study. LBM has proven to be an efficient method to characterize the liquid water transport in the GDL to monitor the impacts of the changes in the capillary pressure and the contact angles of the PTFE contents^24,25^. In a study by Jinuntuya et al.^26^, three different types of GDLs were first analyzed by X-ray computational tomography (CT) followed by analysis based on the LBM. The results showed the critical role of GDL’s material on water management in this layer. The stable movement of water was seen in all the considered GDLs with hydrophilic properties, while capillary fingering was dominating in the hydrophobic conditions. Also, the results indicated that the water invasion patterns and the water saturation do not depend on the applied pressure difference, while they are highly dependent on the GDL microstructure.

Considering the LBM study for the PEMFC, Liao et al.^27^ analyzed the characteristics of the fluid flow in the bipolar plates under air purge using the LBM to improve the design of the flow channel. The electrochemical reaction rates inside the CL were also predicted using LBM by Deng et al.^28^. Results indicated that although higher Pt loading can improve the electrochemical reactions in the CL, it also complicates oxygen transport. The developed LBM study by Wang et al.^29^ delineated that the decreasing of the local capillary pressure in the GDL environment, and the creation of multi-paths GDLs lead to intense flooding problems. The stochastic model by Jiang et al.^30^ investigated the process of melting the ice in cold start conditions and emphasized on the role of carbon fiber diameter to improve water management. A similar stochastic model by Wang et al.^31^ explained the crucial role of having higher PTFE content at the interface of the GDL and the flow channels to ameliorate the water drainage. Using the LBM-based quasi-random nano-structural model of the PEMFC, shin et al.^32^ analyzed different values of the Pt/C catalyst loading and concluded that higher Pt contents in the CL will not improve the reaction rate and it will result in lower pore interconnections, which reduce the catalyst performance and operation. Although different types of stochastic model based on LBM have been developed to characterize the performance of the PEMFC, LBM models based on the obtained real geometry through microscope imaging or CT scan imaging is of interest to have the highest possible accuracy and precision.

Therefore, in a study developed by Sarkezi-Selsky et al.^33^, LBM was used to analyze the transport of liquid water in the GDL using X-ray micro-computed tomography ($\mu$-CT). The results showed as the MPL dominates the capillary transport, the degradation in the MPL has more impact on the water management in PEMFC compared to the degradation of the GDL. Although this study presented interesting results based on the $\mu$-CT images for the pristine samples of the GDL and MPL, the microstructures of the aged samples were not reconstructed by the $\mu$-CT and they were estimated. The basis of the estimation was the enhancement in the porosity of the MPL and the reduction of the PTFE in the GDL. In a similar study, Bosomoiu et al.^34^ used $\mu$-CT images of the GDL to reconstruct the geometry, although they did not differentiate between the carbon fibers and PTFE. It was concluded that the main degradation phenomenon in the GDL is surface hydrophobicity, which was modeled assuming different contact angles that are related to the wettability of the microstructure. The impacts of the PTFE content were also studied by Yu et al.^35^ using the LBM. The results indicated that higher PTFE contents reduce the breakthrough times while enhancing the number of breakthrough locations. Additionally, a valuable study by García-Salaberri et al.^36^ used CT-scan imaging to reconstruct the GDL followed by simulation and experiments to calculate the effective diffusivity^36^ and other microstructural properties such as permeability and electrical/thermal conductivity^37^. Although all the mentioned studies have proposed interesting results to reconstruct the CT-scan images followed by reconstruction to enable the calculation of properties, none of them presented a quantification method to estimate the amount of existing water inside the GDL that may harm the GDL.

**Figure 1:**
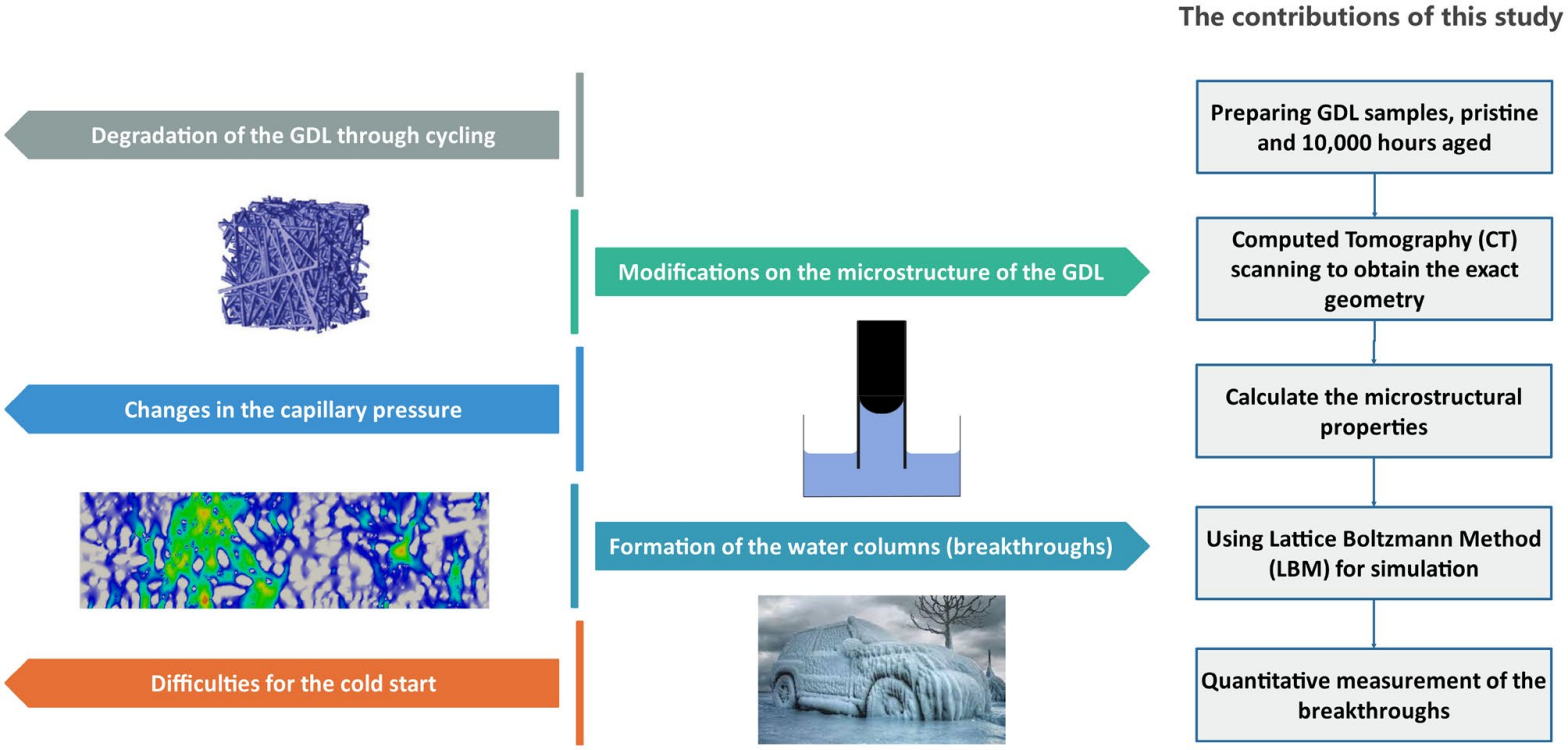
A schematic abstract of the contributions of this study and the main objective of this study.

In this study, an efficient method will be proposed to enable the quantitative measurement of the water columns (breakthroughs) inside the GDL. Two different samples of the GDL, pristine and aged, were characterized using the $\mu$-CT scanning. After imaging, the segmentation and reconstruction were done to prepare the required geometries for fluid flow simulation using the Lattice Boltzmann Method (LBM). Using the obtained images, the microstructural properties of the pristine and aged samples can be obtained to better compare the changes after degradation due to aging. Using LBM and the built geometries, computational fluid dynamic studies were developed to analyze the flow of water considering the capillary pressure inside the GDL. In this regard, the regions, which are prone to have breakthroughs are detected. Considering the pixel values of the breakthrough regions, the quantitative measurement of the water columns became feasible and the corresponding values were reported. Figure 1 presents the schematic flow of the main objectives of this study. The proposed methodology in this study can be a reference for the researchers to characterize the water management capabilities of GDLs before and after experimental tests. This method can be also considered the only possible way to calculate the exact values of the water columns (breakthroughs) inside an arbitrary GDL.

## Problem description

This study aims to propose a reference methodology for the characterization of the water management of different types of GDLs for PEMFC applications. Thus, pristine and aged samples of GDL were scanned using the $\mu$-CT imaging technique to obtain the precise geometry for microstructural and CFD analyses. As the capillary pressure should be considered in the flow field inside the GDL, the CFD analysis was developed using LBM.

In the first step of this study, the definition of the breakthrough should be elaborated, as there have been different definitions in the literature. The accounted breakthrough regions in this study are the deviation of the fluid flow from the homogeneous conditions that will result in the formation of water columns, destruction of the pore structure, and difficulties for the cold start operations.

**Figure 2:**
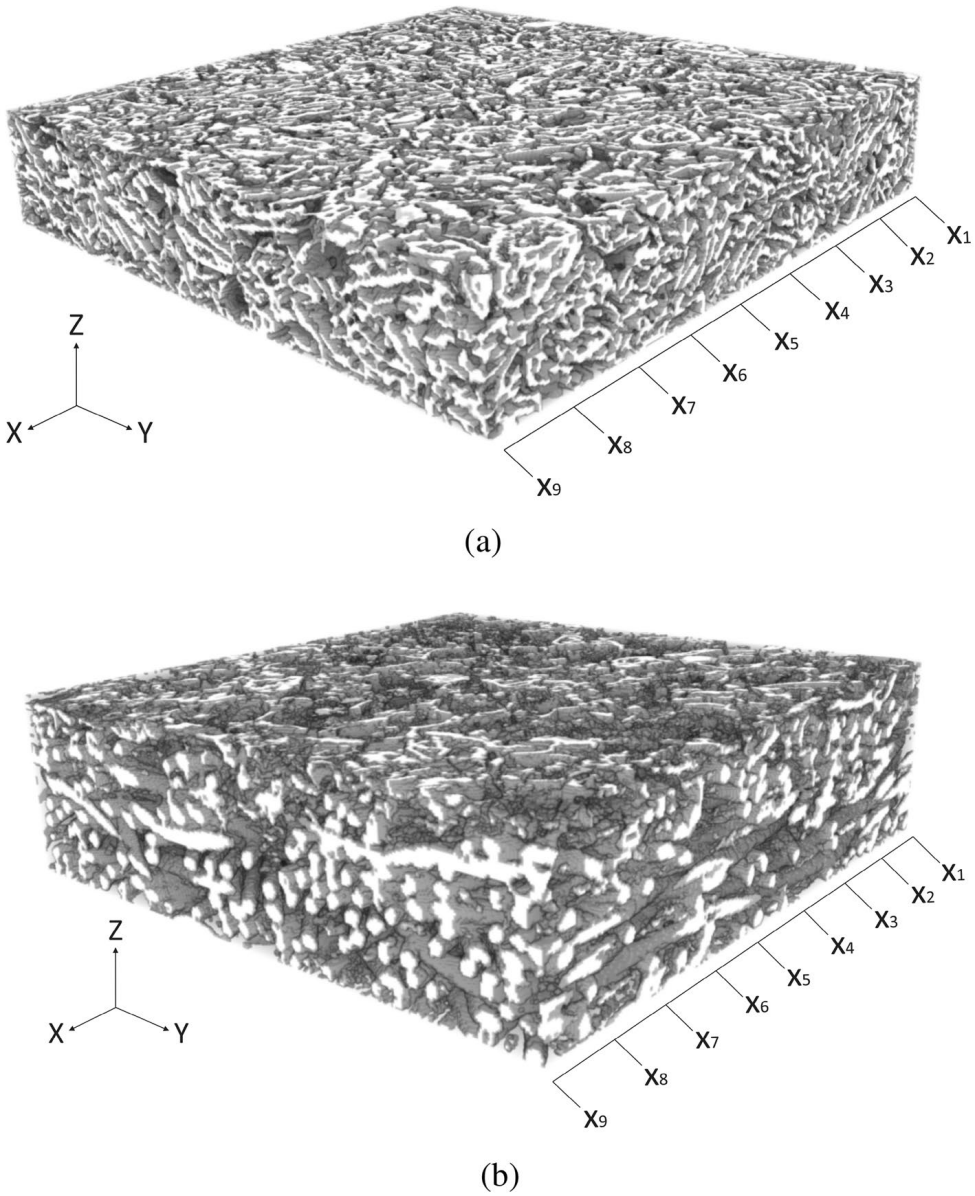
The schematic of the scanned, segmented, and reconstructed GDLs to be analyzed using the LBM: (**a**) The pristine sample, (**b**) The aged sample. The sample and voxel sizes in all the directions are given by Table 1.

Figure 2a,b show $\mu$-CT scan images (after segmentation and reconstruction) for the pristine and aged samples. It should be noted that both pristine and aged GDLs are similar types but one has gone through aging. Although both these samples are the same type, as can be seen in Fig. 2, the structure has been changed in the aged sample compared to the pristine sample. Although the source of this change is degradation through aging, it can be valuable research for future studies to analyze the reason for the appearance of fiber-like structure in the aged sample. The reasoning of the authors is the fact that all the GDLs are made of carbon fibers which are impregnated by PTFE to improve the wettability and contact angle. However, the PTFE becomes degraded through aging and only fibers will remain. The geometry in Fig. 2 has been obtained using $\mu$-CT scan imaging with the operating conditions indicated in Table 1. Both in Fig. 2a,b, the size of the samples are $0.3\times 0.3\times 0.1\hspace{1mm}(\textrm{mm}^3)$, and the image resolution is 1 µm. The segmentation and reconstruction of the images have been done using Dragonfly software, Version 2020.2 developed by Object Research Systems (ORS) Inc in Montreal (Canada). The microstructural properties of the GDLs samples such as connectivity, effective diffusivity, etc. were obtained using in-house codes that were developed in MATLAB.

Once the segmentation and reconstruction of the GDL images are done, the reconstructed models were used to perform the LBM simulation to analyze the water flow in the GDL. The required governing equations to develop the LBM simulation model are presented in “Governing equations” section. The results of the LBM simulation provide the three-dimensional flow distribution of the flow inside the GDL, hence the existence of breakthroughs (water columns) in the GDL can be predicted.

It should be noted that the present study benefits from the same LBM model that was developed in the previous studies of the co-authors^38,39^. In those studies, the solver of the fluid flow simulations was validated with the experimental results of Muljadi et al.^40^. Additionally, the co-authors have compared the LBM results of this model with the Navier-Stokes results in Ref.^41^. It is noteworthy to mention that although the Lattice BGK (Bhatnager-Gross-Krook) model itself does not explicitly include capillary pressure, the developed model by the co-authors benefits from updated collision operators to include the capillary pressure.

**Table 1:** The required parameters to perform the CT scans and LBM study of the analyzed pristine and aged GDL samples.

Parameter	Value
Acceleration voltage	40 kV
Averaging frames	10
Current	120 µA
Exposure time	500 ms
Image resolution	1 µm
Rotation step	0.22 degrees
Sample size, aged (X-direction)	0.3 mm
Sample size, aged (Y-direction)	0.3 mm
Sample size, aged (Z-direction)	0.1 mm
Sample size, pristine (X-direction)	0.3 mm
Sample size, pristine (Y-direction)	0.3 mm
Sample size, pristine (Z-direction)	0.1 mm
Stage temperature	21 Celsius degrees
Voxel size, aged (X-direction)	350
Voxel size, aged (Y-direction)	350
Voxel size, aged (Z-direction)	100
Voxel size, pristine (X-direction)	350
Voxel size, pristine (Y-direction)	350
Voxel size, pristine (Z-direction)	100

## Governing equations

In this section, an overview of the Lattice Boltzmann Method (LBM) and the required formulations to develop the fluid flow analysis are described. Additionally, the needed procedure to obtain the microstructural properties using the CT scans is explained.

Both LBM and Navier-Stokes Equations (NSE) result in the simulation of the fluid flow in a porous structure. In comparison to the NSE, LBM can be even more difficult to solve, but it can consider the impacts of the capillary pressure at the interfaces of different phases.

In LBM, the discrete-velocity distribution function $f_i(x,t)$, which is also called the particle population, plays a key role in solving the problem at the position *x* and the time of *t*. $f_i(x,t)$ is a representative of the particles’ density and velocity, $c_i=(c_{ix},c_{iy},c_{iz})$, as a function of time and space with the squared lattice spacing of $\Delta x$ and time steps of $\Delta t$, respectively.

The mentioned parameters of $\Delta t$ and $\Delta x$ can be considered in any sets of units such as SI or Imperial. However, the common unit in the literature for LBM problems is the Lattice units, which is a simplified set by assuming $\Delta t=1$ and $\Delta x=1$. Regarding the velocities, the discrete velocities, $c_i$, and the weighting coefficients, $w_i$, create velocity sets of $[c_i,w_i]$ that can be presented in the form of DdQq. Here, *q* is the number of velocities of a set and *d* is the velocity set’s number of spatial dimensions. This study uses a D3Q19 velocity set with the velocity components of $[c_{i\alpha }]$ and the weights of $[w_i]$ to enable the three-dimensional simulation of the fluid flow. In each velocity set, the parameter $c_s$, which indicates the isothermal model’s speed of sound, will be included to make a relation between the density $\rho$ and pressure *p* as of $p=c_{s}^{2}\rho$.

Considering the above-mentioned definitions, the discretized form of the Boltzmann equation in the velocity, time, and physical spaces will be as follows:

1 $$\begin{aligned} f_i(x+c_i \Delta t, t+\Delta t)=f_i(x,t)+\Omega _i(x,t) \end{aligned}$$

**Figure 3:**
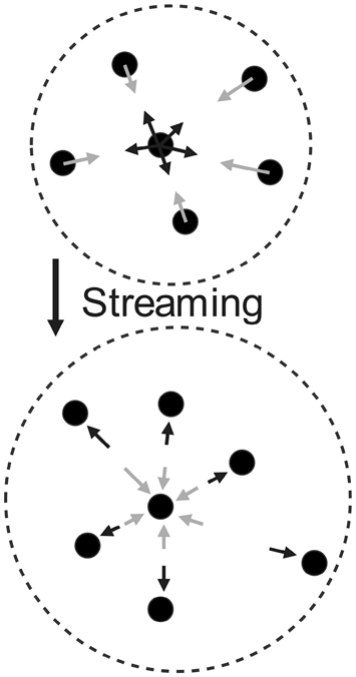
The schematic of the particles’ streaming to their neighbor particles. The central nodes are shown in black and the streamed nodes are in grey. The first (above) picture shows the post-collision distributions $f_{i}^{*}$ while the second (bottom) picture illustrates the pre-collision distributions $f_i$.

Equation (1) denotes the movement of the particles $f_i(x,t)$ to the points $(x+c_i \Delta t)$ at the time step of $(t+\Delta t)$ with the speed of $c_i$ (see Fig. 3). In the same time step, the particles are experiencing collisions with the collision operator ($\Omega _i$).

Although different collision operators can be used, the selected one that is common for fluid flow simulations is the Bhatnagar-Gross-Krook (BGK) operator^42^ as follows:

2 $$\begin{aligned} \Omega _i(f)=-\frac{f_i-f_{i}^{eq}}{\tau }\Delta t \end{aligned}$$

Equation (2) represents that the equilibrium of $f_{i}^{eq}$ can be reached with the defined rate by the relaxation time, $\tau$. Equation (3) presents the required formulation to calculate the $f_{i}^{eq}$:

3 $$\begin{aligned} f_{i}^{eq}(x,t)=w_i \rho \left( 1+\frac{u.c_i}{c_{s}^{2}}+\frac{{(u.c_i)}^2}{2c_{s}^{4}}-\frac{u.u}{2c_{s}^{2}}\right) \end{aligned}$$

The Chapman-Enskog analysis^43^ can be used to make a connection between the Lattice Boltzmann and Navier-Stokes equations. In this regard, the results of these two sets of equations would be similar in macroscopic scale using the kinematic shear viscosity, which is obtained by $\tau$:

4 $$\begin{aligned} \nu =c_{s}^{2}\left( \tau -\frac{\Delta t}{2}\right) \end{aligned}$$

while kinematic bulk viscosity is $\nu _B = 2\nu /3$ and the viscous stress tensor is demonstrated as follows:

5 $$\begin{aligned} \sigma _{\alpha \beta } \approx -\left( 1-\frac{\Delta t}{2 \tau }\right) \Sigma _i c_{i\alpha }c_{i\beta }f_{i}^{neq} \end{aligned}$$

here, $f_{i}^{neq}=f_i-f_{i}^{eq}$ presents the deviation of the $f_i$ from the equilibrium. Using the BGK collision operator given by Eqs. (2) in (1) results in the Lattice BGK (LBGK) equation as follows:

6 $$\begin{aligned}{} & {} f_i(x+c_i \Delta t, t+\Delta t)=\nonumber \\{} & {} \quad \hspace{20mm}f_i(x,t)-\frac{\Delta t}{\tau }[f_i(x,t)-f_{i}^{eq}(x,t)] \end{aligned}$$

Using Eq. (6), two different decompositions of collision and streaming can be obtained. The collision or relaxation can be computed using Eq. (7):

7 $$\begin{aligned} f_{i}^{*}(x,t)=f_i(x,t)-\frac{\Delta t}{\tau }[f_i(x,t)-f_{i}^{eq}(x,t)] \end{aligned}$$

that can be simplified since $\tau /\Delta t =1$ when $f_{i}^{*}(x,t)=f_{i}^{eq}(x,t)$ as follows:

8 $$\begin{aligned} f_{i}^{*}(x,t)=f_i(x,t)\left( 1-\frac{\Delta t}{\tau }\right) +f_{i}^{eq}(x,t)\frac{\Delta t}{\tau } \end{aligned}$$

The streaming or propagation (see Fig. 3) can be also calculated using Eq. (9):

9 $$\begin{aligned} f_i(x+c_i \Delta t, t+\Delta t)=f_{i}^{*}(x,t) \end{aligned}$$

In the first step to implement the LBM, the time should be considered zero, $t=0$, for the density and the velocity. This assumption results in the following equation:

10 $$\begin{aligned} f_{i}^{eq}(x,t=0)=f_{i}^{eq}(\rho (x,t=0),u (x,t=0)) \end{aligned}$$

**Figure 4:**
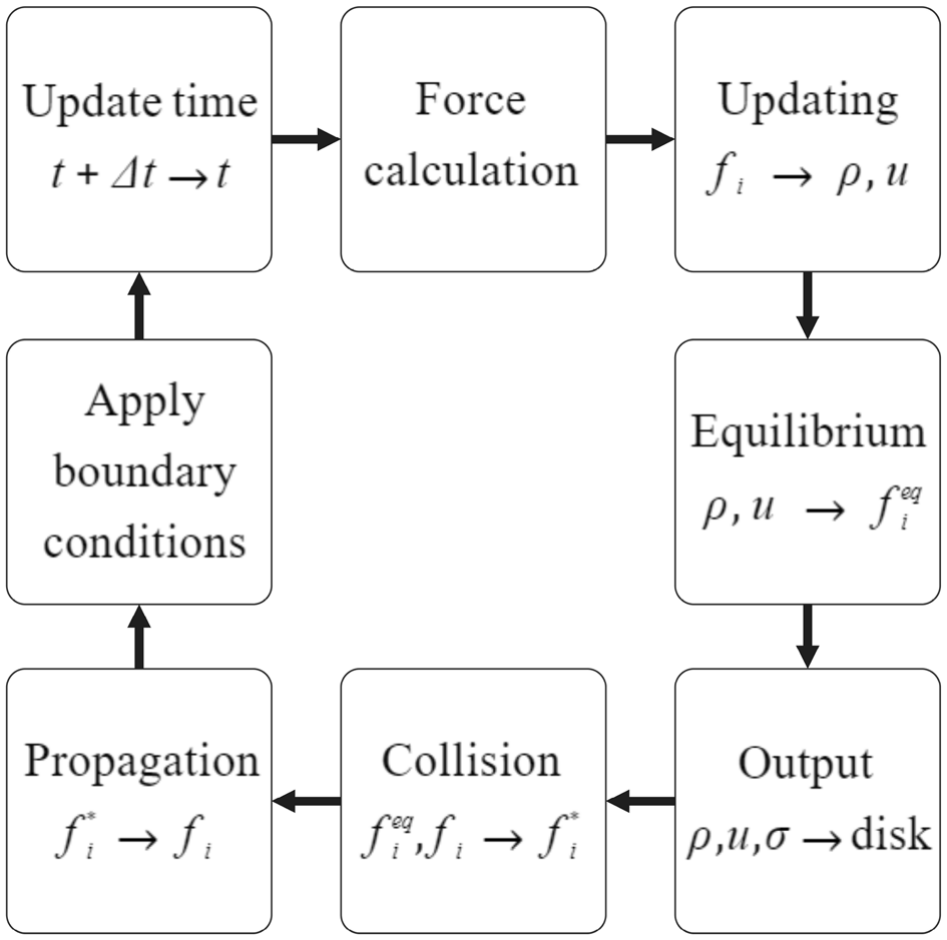
The required algorithm to use the LBM for the simulation of the fluid flow inside the GDL.

Afterward, the suggested algorithm shown in Fig. 4 can be utilized as follows:

- Calculating the density, $\rho (x,t)$, and velocity, *u*(*x*, *t*), using the $f_i(x,t)$.
- Determining the $f_{i}^{eq}(x,t)$ using Eq. (3).
- Computing the $\sigma$ using Eq. (5).
- Developing the collision utilizing Eq. (9).
- Streaming can be achived by Eq. (10).
- Progressing to the next time step if the convergence is not achieved.

### The calculation of the microstructural properties

The measurement of the metric properties (i.e., volume fractions, surface contact areas, and mean phase diameters), in addition to the connectivity, is performed using in-house routines with the details given by ref.^44^.

The quantification of the diffusive transport in GDL is done by solving the partial differential equation. It is assumed that the heat fluxes can be modeled by Fourier’s law, the electron fluxes by the local Ohm’s law, and the diffusion fluxes by Fick’s law. Thus, the stationary heat equation models the diffusion of the Oxygen and water vapor in the gas phase, the conduction of heat in the solid and gas phases, and the electrical conduction in the solid phase.

The heat equation can be obtained using Fourier’s law. In the first step, the heat flux $\bar{q}$ should be calculated using the temperature gradient:

11 $$\begin{aligned} \bar{q}=\beta \bar{\nabla } T \end{aligned}$$

where, *T*(*K*) is the temperature, $\beta (\bar{x}) (\frac{W}{m.K})$ is the local thermal conductivity at point $\bar{x}=(x_1,x_2,x_3)$, and $\bar{\nabla }$ is the divergence operator.

A balance of energy leads to the equation of heat in the material:

12 $$\begin{aligned} \bar{\nabla }.(\beta \bar{\nabla } T)=\rho c \frac{\partial T}{\partial t} \end{aligned}$$

Where $\rho (\frac{\textrm{kg}}{\textrm{m}^3})$ is the density of the material, and *c* is the specific heat of the material in $(\frac{J}{kg.K})$. Equation (13) presents the heat equation in a steady-state condition:

13 $$\begin{aligned} \bar{\nabla }.(\beta \bar{\nabla } T)=0 \end{aligned}$$

Several simplifications are made when the diffusion of gas and the conduction of electricity and heat are modeled by the stationary heat equation. For diffusion, this is equivalent to modeling the relationship between flux and concentration by Fick’s law rather than by Stefan-Maxwell’s law. Thus, the diffusion effects in ternary gas mixtures are neglected. It is also assumed that the binary diffusion coefficient does not depend on the concentration. Concerning the heat transfers, only the conduction is considered and the radiation heat transfers are not taken into account similar to thermal expansion. Conducting effects appearing at the nanoscale are not considered, for example, impedance mismatches of phonons in the case of heat transfer, or conduction of electrons on the surface for electrical transfers. These effects can reasonably be considered as negligible at the micro-metric scales.

## Results and discussion

### Microstructural properties

**Figure 5:**
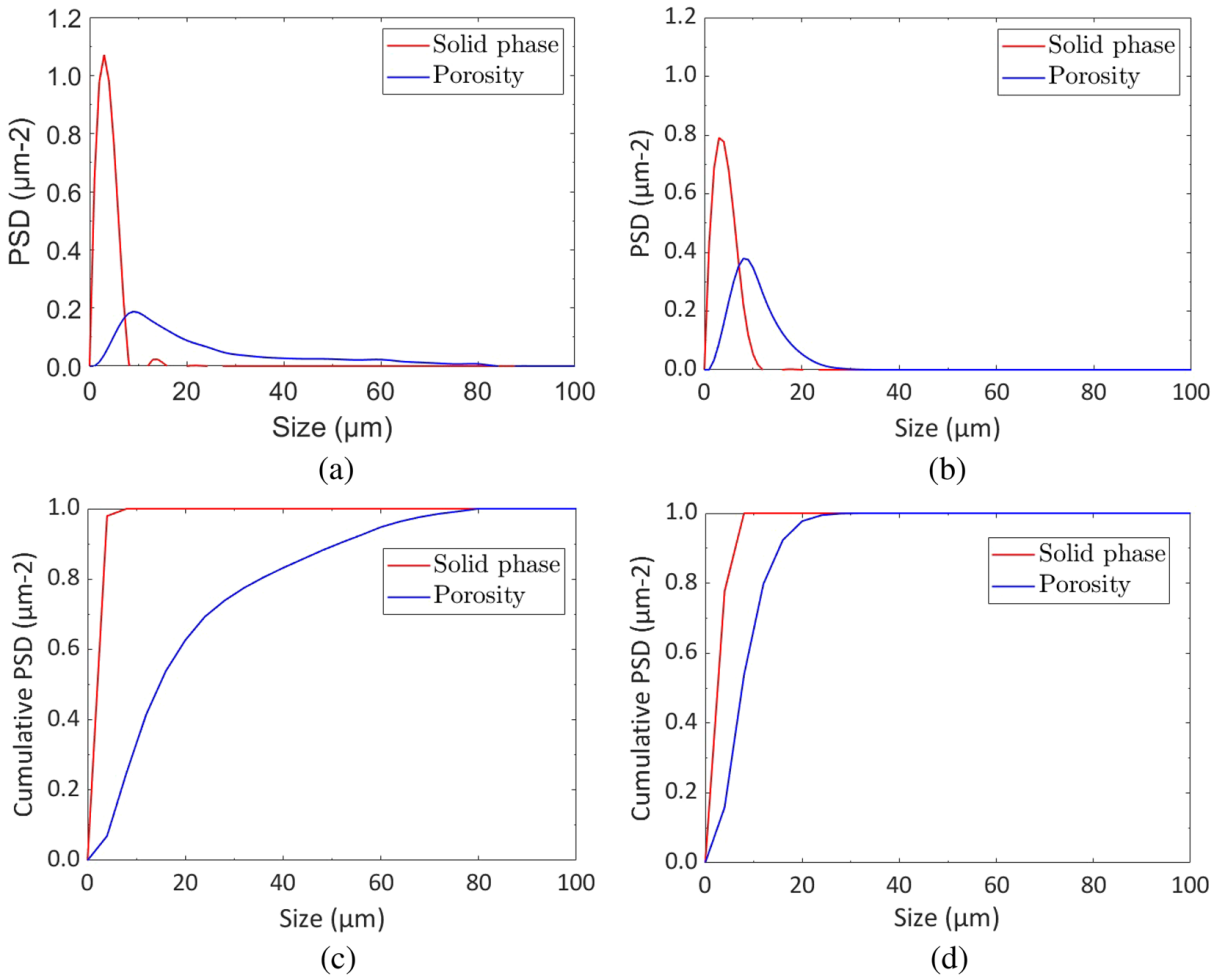
The comparison between the particle size distribution (PSD) of the pristine and aged GDLs: (**a**) The PSD of the pristine sample, (**b**) The PSD of the aged sample, (**c**) The cumulative PSD of the pristine sample, (**d**) The cumulative PSD of the aged sample.

For the CFD analysis of the shown GDL samples in Fig. 2, the samples should be scanned with $\mu$-CT imaging to enable the segmentation and reconstruction of the exact geometry. However, the LBM simulation demands the calculation of the microstructural properties such as porosity and the mean diameter as they can be considered as boundary conditions. Table 2 presents the obtained microstructural properties using the given governing equations in “Governing equations” section. Figure 5 also delineates the changes in the particle size distribution (PSD) and the cumulative PSD by the variation of the sizes in the porous and solid regions.

**Table 2:** The obtained microstructural properties of the GDL samples.

Region	Connectivity (%)	Normalized effective diffusivity (–)	Mean diameter (µm)	Surface contact area ($\frac{\upmu {\rm m}^2}{\upmu {\rm m}^3}$)	Volume fraction (%)
Porous, aged	100	0.86	14.76	7.67	77.42
Porous, pristine	100	0.76	7.6	8.33	70.33
Solid, aged	93	0.17	2.06	7.67	22.58
Solid, pristine	93	0.26	2.56	8.33	29.67

The primary microstructural change evident in Table 2 is the substantial enhancement, nearly doubling, of the mean diameter for the porous regions after aging, increasing from 7.6 to 14.76 µm. In contrast, the mean diameter for the solid region experiences a slight reduction from 2.56 to 2.06 µm. The shift in phase size can be attributed to corrosion, the removal of the binder, or the carbon fibers during the cycling processes. This hypothesis aligns with the observed reduction in the volume fraction of the solid phase, decreasing approximately by 7% from the pristine to the aged sample.

Considering the given parameters in Table 2, the surface contact area refers to the total surface area per unit volume of a porous material. It quantifies the available surface for interactions such as adsorption, chemical reactions, and fluid flow. The volume fraction in porous regions refers to the proportion of the total volume where fluid flow effectively occurs. Regarding the mean diameter, a porous medium consists of pores between particles, contained within a vessel or control volume. In this study, the mean diameter is defined as the diameter of a sphere with the same volume-to-surface area ratio as the actual particle.

Regarding transport properties, as anticipated, the aforementioned microstructural changes lead to an improvement in pore phase diffusivity, changing from 0.76 to 0.86 and from 0.26 to 0.17, respectively. It’s important to note that values close to 1 represent better transport properties. The combined effect of changes in phase mean diameter and volume fraction maintains the surface area of the two phases relatively constant. It should be also mentioned that the effective diffusivity was calculated by dividing the intrinsic diffusivity by porosity and tortuosity. Tortuosity refers to the winding or convoluted path that matter (such as fluids or solutes) takes when moving through a porous substance. The effective diffusivity in this context means the reduced intrinsic diffusivity by the existing pore structure and the tortuosity^45^.

Phase connectivity in porous media refers to how different fluid and solid phases are interconnected within the pore space of a porous material. In terms of phase connectivity, the aging appears to significantly alter neither the porous nor the solid phase, with high values remaining at 100% and 93%, respectively. Furthermore, Fig. 5a,b indicate that the maximum PSD of the solid phase is reduced from around 1.08–0.8 µm^−2^ followed by the enhancement in the maximum value of the PSD for the porous region from around 0.2–0.4µm^−2^. The comparison between Fig. 5c,d shows that the porous region in the pristine sample and a smoother increase of the cumulative PSD by the changes in the size, which indicate the existence of small porous regions in the domain that prevent the capillary fingering and the formation of the breakthroughs in the medium. However, Fig. 5d shows that the enhancement of the cumulative PSD in the porous region is sharp, hence the possibility of breakthrough formation inside the domain.

### Fluid flow simulation using LBM

Among the existing methods to perform the LBM simulations, Single-Relaxation Times (SRT), Two-Relaxation Times (TRT), or Multi-Relaxation Time (MRT) are available. This study is using the MRT-LBM, as mentioned in “Governing equations” section to simulate the fluid flow inside the scanned GDL sample by the $\mu$-CT imaging. Each of the lattice vectors is located in the position of *x* and the time of *t*, which enables the time-dependent simulation of the fluid flow inside the GDL. In this study, the D3Q19 discretization model has been used, which means each of the lattice vectors has 19 different components in the momentum space.

It should be mentioned that the boundary conditions of this study are a low-speed (laminar flow) fluid flow and the Darcy condition regarding the flow inside a porous medium^46^. As the capillary pressure is one of the main driving forces, the LBM model also takes into account this pressure in addition to the pressure gradient, which is 0.1 $\frac{Pa}{m}$ in the Z-direction of both samples that are shown in Fig. 2. The surfaces parallel to the ZY and XZ planes in Fig. 2 have the bounce-back boundary condition, which is considered a simple method to locate a no-slip fluid flow halfway between a wall and a fluid.

To simplify the fluid flow inside the considered aged and pristine GDL sample, the working fluid is accounted to be pure water with the kinematic viscosity of 0.802 $\frac{\textrm{mm}^2}{s}$ and the fluid density of 995.7 $\frac{\textrm{kg}}{\textrm{m}^3}$. In reality, the hydrogen gas in the anode and the oxygen from the air in the cathode, flow inside the GDL to be diffused in a humid condition to be prepared for the electrochemical reactions in the catalyst layers. In the real application of the PEMFC, the existing humidity in the GDL results in the formation of water droplets that may stay in the GDL and also flow in this medium. In this regard, the assumption of having pure water as the working fluid denotes 100% flooding of the cell and can be a good assumption to examine the water removal capability of the considered GDLs.

To perform the LBM simulation, the maximum number of iterations for flow simulation is considered to be 80,000,000 while the Navier-Stokes relaxation time is 1. Additionally, the frequency that the convergence being checked is 0.5 *s* while the convergence criteria for the flow field is $10^{-4}$. The inflation parameter regarding the LBM simulation, which considers the surface mesh before the visualization is 0.1. These parameters are selected similarly for both the pristine and aged samples to eliminate the possible deviations in the results.

**Figure 6:**
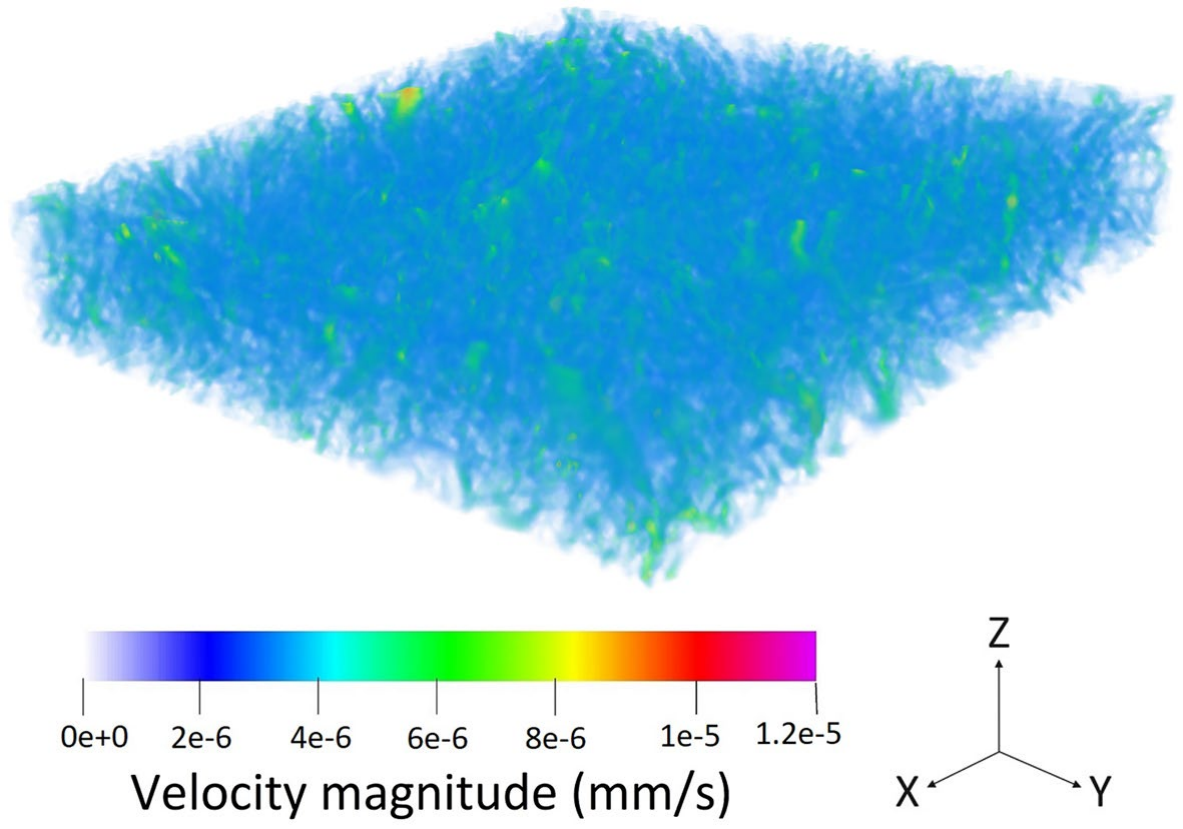
The three-dimensional velocity contours in the pristine GDL sample with the geometry given by Fig. 2a.

**Figure 7:**
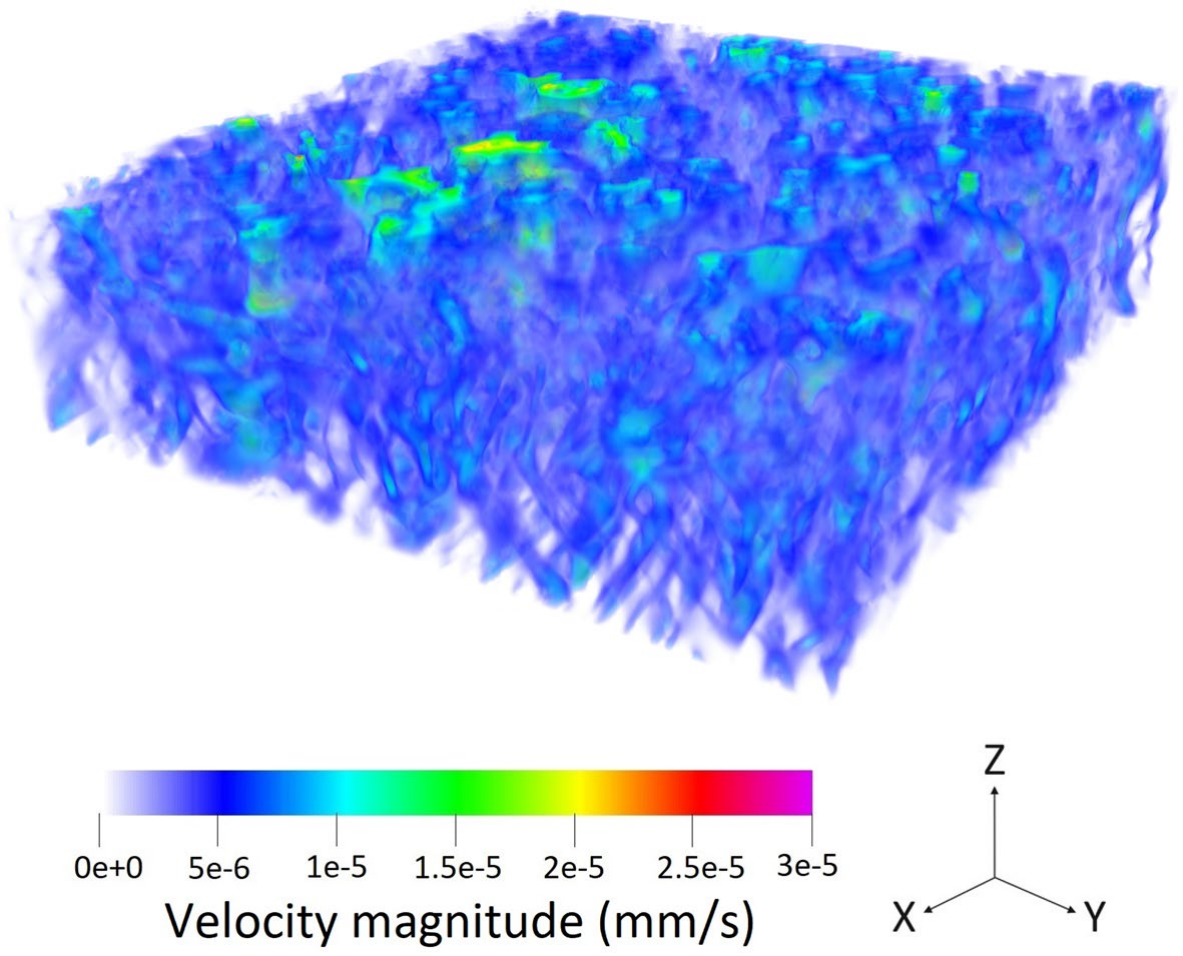
The three-dimensional velocity contours in the aged sample with the geometry given by Fig. 2b.

**Figure 8:**
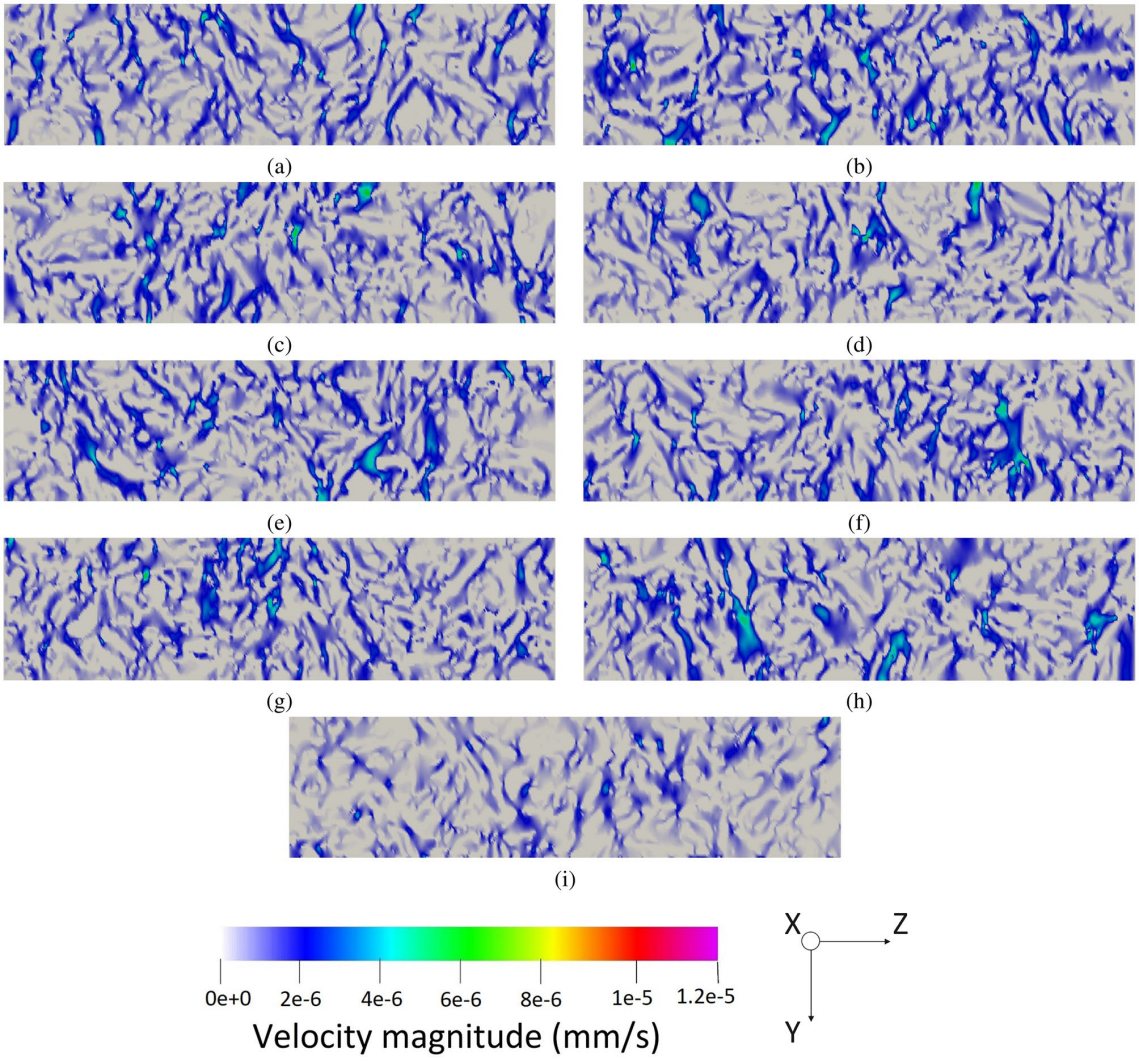
The two-dimensional illustration of the velocity contours in the pristine sample, obtained by the LBM simulation, based on the defined planes in Fig. 2a: (**a**) $X_1$, (**b**) $X_2$, (**c**) $X_3$, (**d**) $X_4$, (**e**) $X_5$, (**f**) $X_6$, (**g**) $X_7$, (**h**) $X_8$, (**i**) $X_9$.

**Figure 9:**
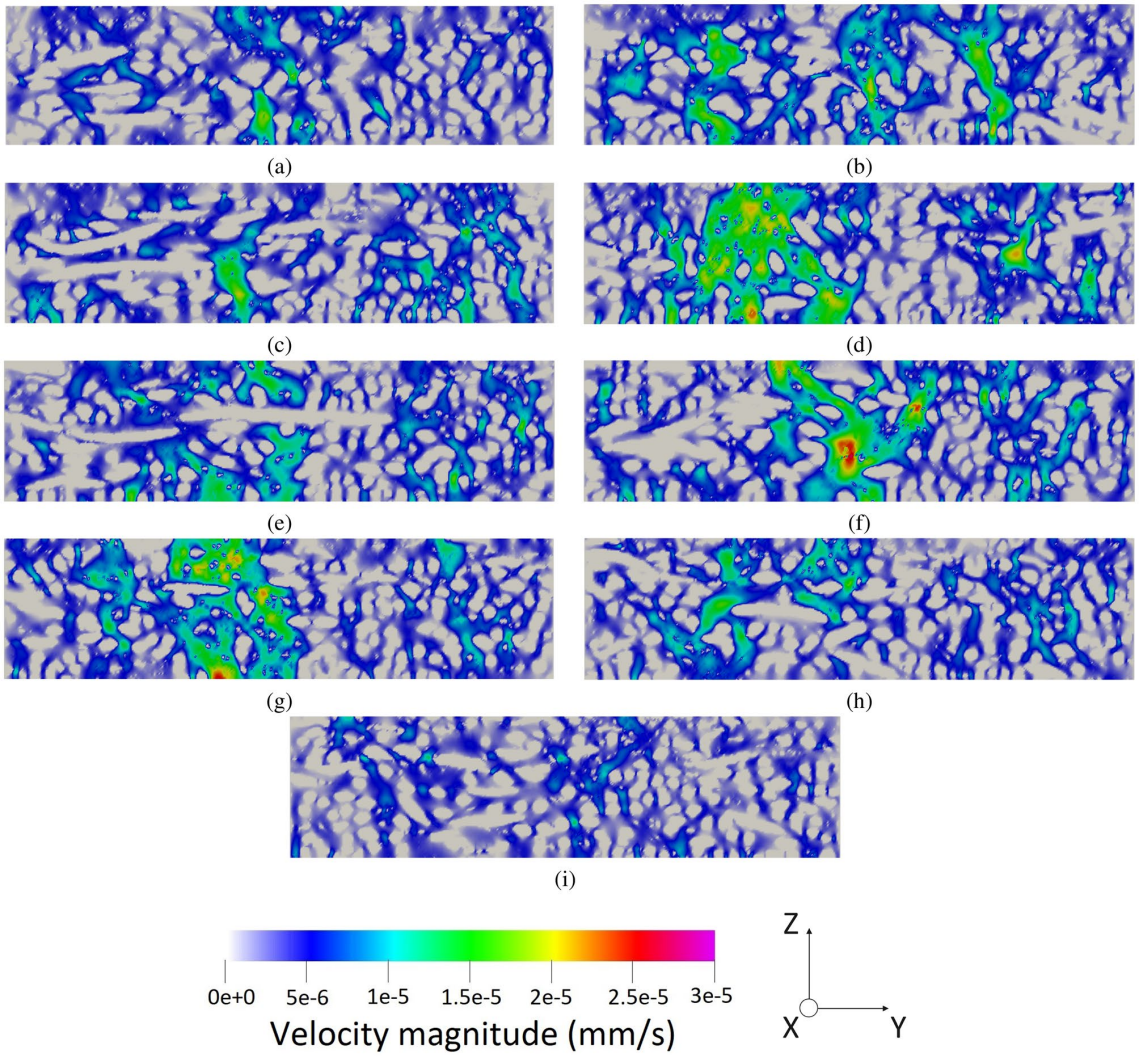
The two-dimensional illustration of the velocity contours in the aged sample, obtained by the LBM simulation, based on the defined planes in Fig. 2b: (**a**) $X_1$, (**b**) $X_2$, (**c**) $X_3$, (**d**) $X_4$, (**e**) $X_5$, (**f**) $X_6$, (**g**) $X_7$, (**h**) $X_8$, (**i**) $X_9$.

As all the boundary conditions, operating parameters, and domains are defined, the simulation run can be done. Figure 6 illustrates the three-dimensional fluid flow in the pristine GDL sample, which is shown in Fig. 2a while Fig. 7 delineates a similar three-dimensional illustration for the aged sample shown by Fig. 2b. To better visualize the fluid flow inside the carbon fibers and the binder of the porous region, nine different slices, namely, $X_1$, $X_2$, $X_3$, $X_4$, $X_5$, $X_6$, $X_7$, $X_8$, and $X_9$, are selected based on Fig. 2a and the corresponding two-dimensional contours are shown in Fig. 8 for the pristine sample. Similarly, the selection of the nine different slices has been done using Fig. 2b for the aged sample, and the fluid flow in those slices is shown in Fig. 9. The regions with higher velocities, as demonstrated by the velocity bar below the figures, have red, yellow, and green colors, which indicate the vulnerability for the creation of the water columns (breakthroughs) inside the GDL.

The concept behind this section is that velocity or any other simulation result is not a parameter to characterize the real exact behavior inside the GDL. Microstructure or wettability cannot be quantified to enable comparison of different GDLs. In this regard, the only solution so far was to test the GDLs experimentally in stack testing facilities and compare the characteristic curves such as I-V curve. The novelty of this study is to propose a new approach to enable simulation comparison of GDLs. The degrading phenomena in GDL related to the water is not the velocity but rather the water accumulation in the GDL, which will lead to difficulties during startup and the destruction of GDL’s pore structure. In this case, the water should be distributed homogeneously and the velocity contours should indicate the uniform distribution of flow inside the GDL. The accounted breakthrough regions are not selected based on the velocity but rather the deviation from the homogeneous condition. In this case, the uniform condition should benefit from a certain average velocity, and the deviation from this speed is considered a breakthrough region. In the pristine sample, the homogeneous average speed of flow should be around 2 nm/s while in the aged sample, it should be about 5 nm/s based on the observed microstructure using CT scan imaging. Lower velocities than these values are the pore structure of the GDL or the boundary layers on the surface, however, higher velocities are an indication of deviation from the homogeneous conditions which will increase the possibility of water accumulation inside the GDL.

Regarding the obtained two-dimensional contours of the fluid flow inside the GDL, which are illustrated in Figs. 8 and 9, although the visual observation of the breakthroughs inside the pristine and aged GDL samples may be possible, better methods should be used to facilitate the quantitative measurement and comparison of the breakthroughs. In this regard, each voxel that has velocities higher than $4.5\times 10^{-9} \frac{\textrm{m}^3}{s}$ in Fig. 8 and $1.2\times 10^{-8} \frac{\textrm{m}^3}{s}$ is accounted as a breakthrough region with the possibility of degrading the cell and the GDL. Considering this assumption, 6.32% of the pristine GDL sample shown in Fig. 2a and 10.12% of the aged GDL sample shown in Fig. 2b are prone to have breakthroughs in a real application, which will fill the GDL pores and degrades PEMFC’s operation. As it was mentioned that each sample has an overall size of 12,250,000 µm^3^, this means that breakthrough regions in the pristine and aged GDL samples are 774,200 µm^3^ and 1,239,700 µm^3^, respectively.

## Conclusion

The aim of the current study was to analyze the water formation in the gas diffusion layer (GDL) and compare the differences between the pristine and aged GDL samples. The formation of water columns, which is also famous as breakthroughs, is known as a degradation phenomenon that fills the pores and leads to difficulties in starting the cell from sub-zero temperatures. As the driving force of the fluid flow in the GDL is the capillary pressure, the conventional conservation principles such as momentum, energy, and mass are not the governing equations and methods based on the kinetics of the particles should be used. In this regard, LBM formulation, which is considered a powerful CFD methodology based on the kinetics of the particles to simulate the fluid flow at the interfaces between the gas/solid/liquid and at low-velocity conditions, has been used using the obtained $\mu$-CT images of the GDL.

The $\mu$-CT images were then analyzed to calculate the microstructural properties such as the mean diameter, effective diffusivity, connectivity, surface contact area, and volume fraction. The results of the microstructural study revealed that due to the degradation of the GDL sample or the removal of the binder after aging, the corresponding values of the mean diameter and volume fraction will be reduced for the solid phase, hence bigger regions will be dedicated to the porous media. The effective diffusivity in the porous region will be also reduced due to the aging of the GDL.

Using the exact geometries of the GDL samples that were obtained by $\mu$-CT images, the LBM simulations were performed to calculate the velocities of the water passing through the GDL. The illustrated contours visualized the locations of the breakthroughs followed by the corresponding voxels. To enable the quantitative measurement of the breakthroughs, the voxels with higher velocity values of $4.5\times 10^{-9} \frac{\textrm{m}^3}{s}$ in the pristine sample and higher velocity values of $1.2\times 10^{-8} \frac{\textrm{m}^3}{s}$ in the aged sample are considered as the breakthrough region with the possibility of water column formation. In this regard, 6.32% of the pristine GDL sample and 10.12% of the aged GDL sample are prone to have breakthroughs. As it was mentioned that each sample has an overall size of 12,250,000 µm^3^, this means that breakthrough regions in the pristine and aged GDL samples are 774,200 µm^3^ and 1,239,700 µm^3^, respectively.

The output results of this study proved that the performance of the GDLs can be characterized without experimental testing and novel GDLs can be analyzed before assembly to reduce the costs and time. The current novelty of this study can be used in future studies to facilitate GDL characterization. Additionally, the following topics can be accounted for future studies:

- This study has only considered the simple operation of the fuel cell after aging and used the corresponding GDL for the water management analysis. However, there are different types of degradation phenomena in the PEMFC, namely chemical oxidation, electrochemical carbon corrosion, freezing/thawing, mechanical degradation, material dissolution, and erosion by gas flow that can be considered. The utilized GDLs in these degradation tests can be later used to compare the changes in the breakthrough regions.
- To facilitate the LBM simulations, simplifications were made and only pure water was considered as the fluid flow in the GDL. However, in reality, the fluid is either hydrogen or oxygen in a humid environment with the possibility of water formation.
- Although the presented aged and pristine GDLs in Fig. 2 are the same type, the structure of the aged GDL has been changed compared to the pristine sample. Although the source of this change is degradation through aging, it can be valuable research for future studies to analyze the reason for the appearance of fiber-like structure in the aged sample.

## Data Availability

The datasets used and/or analysed during the current study available from the corresponding author on reasonable request.
